# High‐Resolution Infrared Synchrotron Investigation of (HCN)_2_ and a Semi‐Experimental Determination of the Dissociation Energy *D*_0_

**DOI:** 10.1002/cphc.201900811

**Published:** 2019-11-08

**Authors:** D. Mihrin, P. W. Jakobsen, A. Voute, L. Manceron, R. Wugt Larsen

**Affiliations:** ^1^ Department of Chemistry Technical University of Denmark Kemitorvet 206 2800 Kgs. Lyngby Denmark; ^2^ Synchrotron SOLEIL L'Orme des Merisiers Saint-Aubin-BP 48 91192 Gif-sur-Yvette Cedex France; ^3^ Lab. MONARIS CNRS-UPMC UMR8233 4 Place Jussieu 75230 Paris Cedex France

**Keywords:** Dissociation Energy, Hydrogen Bonding, Infrared Synchrotron Radiation, Non-Covalent Forces, Vibrational Zero-Point Energy

## Abstract

The high‐resolution infrared absorption spectrum of the donor bending fundamental band *ν* ${}_{6}^{1}$ of the homodimer (HCN)_2_ has been collected by long‐path static gas‐phase Fourier transform spectroscopy at 207 K employing the highly brilliant 2.75 GeV electron storage ring source at Synchrotron SOLEIL. The rovibrational structure of the *ν* ${}_{6}^{1}$ transition has the typical appearance of a perpendicular type band associated with a Σ–Π transition for a linear polyatomic molecule. The total number of 100 assigned transitions are fitted employing a standard semi‐rigid linear molecule Hamiltonian, providing the band origin *ν*_0_ of 779.05182(50) cm^−1^ together with spectroscopic parameters for the degenerate excited state. This band origin, blue‐shifted by 67.15 cm^−1^ relative to the HCN monomer, provides the final significant contribution to the change of *intra*‐molecular vibrational zero‐point energy upon HCN dimerization. The combination with the vibrational zero‐point energy contribution determined recently for the class of large‐amplitude *inter*‐molecular fundamental transitions then enables a complete determination of the total change of vibrational zero‐point energy of 3.35±0.30 kJ mol^−1^. The new spectroscopic findings together with previously reported benchmark CCSDT(Q)/CBS electronic energies [Hoobler *et al. ChemPhysChem*. **19**, 3257–3265 (2018)] provide the best semi‐experimental estimate of 16.48±0.30 kJ mol^−1^ for the dissociation energy *D*_0_ of this prototypical homodimer.

## Graphical abstract

**The rotationally resolved** infrared spectrum of the hydrogen bond donor bending fundamental transition ν${}_{6}^{1}$ of (HCN)_2_ has been observed by long‐path static gas‐phase Fourier transform spectroscopy at 207 K employing the highly brilliant 2.75 GeV electron storage ring source at Synchrotron SOLEIL.

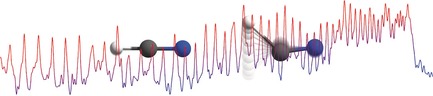

## 1 Introduction

A highly accurate experimental determination of the dissociation energy *D*_0_ for a binary non‐covalent weakly bound cluster molecule is notoriously challenging and has solely been demonstrated for a small number of molecular systems. In the elementary case of the simple hydrogen‐bonded (HF)_2_ homodimer, direct measurements of unparalleled accuracy based on state‐to‐state vibrational pre‐dissociation dynamics by Miller *et al*.^1^ provided a *D*_0_‐value of 1062±1 cm^−1^. In a fully non‐empirical quantum chemical computational work, Hobza *et al*.^2^ employed large basis set CCSD(T) calculations including contributions of higher excitations up to the full CCSDTQ level and relativistic and diagonal Born‐Oppenheimer corrections together with anharmonic vibrational zero‐point energies in order to reproduce the dissociation energy. This demanding computational approach solely available for molecular systems of a very limited size, however, still underestimated the experimental dissociation energy by 25 cm^−1^ owing to the inaccuracy of the second order vibrational perturbation theory approach employed to predict the vibrational zero‐point energy of the (HF)_2_ system. In a similar direct fragment analysis based on velocity map imaging and resonance‐enhanced multiphoton ionization, Rocher‐Casterline *et al*. provided an accurate experimental dissociation energy of 1105±10 cm^−1^ for the slightly larger prototypical hydrogen‐bonded (H_2_O)_2_ homodimer.^3^ This experimental determination helped to validate the accuracy of different theoretical methodologies and most notably the comprehensive work by Shank *et al*. who constructed an intermolecular potential energy surface (IPES) based on 30 000 *ab initio* CCSD(T) grid points.^4^ This full‐dimensional IPES was fitted to reproduce available benchmark calculations of the interaction energy *D_e_* 5 and employed for diffusion Monte Carlo calculations of the vibrational zero‐point energy of (H_2_O)_2_ to predict a dissociation energy *D*_0_ of 1103 cm^−1^. An alternative indirect approach was demonstrated by Kollipost *et al*.^6^ for an even larger system, the doubly hydrogen‐bonded dimer of formic acid (HCOOH)_2_, based on the macroscopic dissociation equilibrium constant and the rich rovibrational spectroscopic datasets available for this strongly bound system. After an extensive far‐infrared jet spectroscopic characterization of large‐amplitude hydrogen bond vibrational modes,^6^, ^7^ the combination of room temperature dissociation equilibrium constants and statistical treatments of the rovibrational partition function involving the complete set of altogether 24 vibrational fundamental transitions enabled the determination of an experimental *D*_0_‐value of 59.5(5) kJ mol^−1^. The present work demonstrates an indirect semi‐experimental strategy for the homodimer of HCN, where an extensive rovibrational dataset for the basically complete set of thirteen fundamental transitions is now available.

The initial spectroscopic investigations of (HCN)_2_ by microwave molecular beam spectroscopy established the linear CH⋅⋅N hydrogen bond configuration in the vibrational ground state.^8^, ^9^, ^11^, ^12^, ^13^, ^14^, ^15^, ^16^, ^17^ Subsequently, complementary high‐resolution infrared^18^, ^19^, ^20^, ^21^, ^22^, ^23^, ^24^ and Raman^25^, ^26^ spectroscopic studies employing a combination of static cryogenic long‐path absorption cells and supersonic jet expansions have provided accurate hydrogen bond induced spectral shifts and (partly) resolved the rovibrational structures of the more or less perturbed intramolecular CH (*ν*_1_ and *ν*_2_) and CN (*ν*_3_ and *ν*_4_) stretching bands of both the hydrogen bond donor and acceptor moieties. The dedicated line shape analysis and extracted line width parameters from the high‐resolution infrared spectra of the hydrogen bond acceptor and donor CH stretching bands have provided crucial information about pre‐dissociation lifetimes and indirectly the coupling between these intramolecular vibrational modes and the large‐amplitude intermolecular hydrogen bond modes of (HCN)_2_.^18^, ^19^, ^23^ Miller *et al*.^20^ generated optothermal sub‐Doppler resolution (near)‐infrared spectra of the *ν*_1_+$\nu_{9}^{1}$ −$\nu_{9}^{1}$ hot band and the *ν*_1_+$\nu_{9}^{1}$ sum band providing indirect information about the doubly degenerate $\nu_{9}^{1}$ fundamental transition associated with the intermolecular large‐amplitude hydrogen bond acceptor librational motion. This $\nu_{9}^{1}$ band origin was subsequently detected directly at 40.7518711(67) cm^−1^ by a tunable far‐infrared Stark spectroscopy investigation.^27^ The observed reduction of the electric dipole moment of 0.54(5) D in the excited state relative to the ground‐state value of 6.023(31) D demonstrated a highly anharmonic nature of this vibrational normal coordinate. The large‐amplitude vibrational motion involving intermolecular hydrogen bonds is in general found to be highly anharmonic in nature and challenging for *ab initio* methodologies.^28^, ^29^, ^30^, ^31^, ^32^ The second fundamental transition associated with the class of large‐amplitude anharmonic intermolecular vibrational modes, the hindered translational motion involving both HCN subunits or intermolecular stretching *ν*_5_, has been observed indirectly at ca. 101 cm^−1^ from vibrational satellites in the microwave region^8^. Recently, the final fundamental transition associated with this class of motion, the doubly degenerate intermolecular large‐amplitude hydrogen bond donor librational mode $\nu_{8}^{1}$ has been observed at 119.11526(60) cm^−1^ by the present authors^33^ employing a high‐resolution long‐path Fourier transform THz spectroscopy approach involving highly brilliant synchrotron radiation.^34^, ^35^, ^36^, ^37^ These experimental rovibrational observations would help to validate or even construct a future (semi‐experimental) full‐dimensional IPES for this prototypical (HCN)_2_ system. In the present work we extend this long‐path synchrotron spectroscopy approach to explore the region above 700 cm^−1^, where the two until now non‐observed vibrational fundamental transitions for (HCN)_2_ associated with the doubly degenerate donor (*ν* ${}_{6}^{1}$ ) and acceptor (*ν* ${}_{7}^{1}$ ) bending modes were expected. In contrast to the acceptor bending fundamental, the donor bending fundamental has been predicted to be significantly blue‐shifted relative to the HCN monomer fundamental at 711.90 cm^−1^ in the order of 55 to 85 cm^−1^ by harmonic^33^ and anharmonic force field calculations^38^, respectively. This doubly degenerate *ν* ${}_{6}^{1}$ transition then alone contribute with ∼0.65–1.0 kJ mol^−1^ to the total change of vibrational zero‐point energy. A recent fully non‐empirical quantum chemical computational work by Hoobler *et al*.^38^ has provided an AE‐CCSDT(Q)/CBS benchmark value including relativistic and diagonal Born‐Oppenheimer corrections for the interaction energy *D_e_* of 19.83 kJ mol^−1^. Instead of employing Hoobler *et al*.’s theoretical anharmonic vibrational zero‐point energy for (HCN)_2_ based on second order vibrational perturbation theory, we are now able to estimate a semi‐experimental value of this important quantity and reach an accurate dissociation energy *D*_0_.

## Experimental

HCN was synthesized by dropwise addition of concentrated H_2_SO_4_ (99.999 %, Sigma Aldrich) onto KCN (≥98.0 %, Sigma Aldrich) *in vacuo* with immediate condensation of the evolved gas. Minor impurities of CO, CO_2_ and (CN)_2_ were subsequently removed by several freeze‐pump‐thaw cycling procedures. A sublimation pressure of 1.7 hPa HCN resided in a static long‐path cryogenic absorption cell at a PID regulated cell body temperature of 207±0.2 K.^39^ The multipass arrangement of the long‐path absorption cell is based on the optical design by Chernin and Barskaya^40^ and provided a total optical path length of 105 m. A specialized transfer optics design is employed to extract and refocus the probe beam onto the sample compartment of a Bruker IFS 125 HR Fourier transform spectrometer (FTS) located at the far‐infrared AILES beam‐line at Synchrotron SOLEIL as described elsewhere.^39^ High brightness broadband synchrotron radiation from the third generation 2.75 GeV electron storage ring providing a ring current of 450 mA was focused onto the aperture of the FTS, providing a signal‐to‐noise gain at high spectral resolution relative to a conventional thermal radiation source.^41^ A total number of 1408 sample single‐beam interferograms, corresponding to a total scan time of 34 hours, was collected employing a Ge on KBr beam splitter and a highly sensitive home‐built liquid helium cooled HgCdTe detector mounted with a cold 940 cm^−1^ low‐pass filter.^42^ The recorded sample interferograms were Fourier transformed employing Mertz phase correction and boxcar apodization. A sample spectral resolution of 0.004 cm^−1^ was selected as the best compromise between the resulting signal‐to‐noise and the separation of observed spectral features. The background single‐beam spectra were collected at a lower but still sufficient spectral resolution to capture the dominant interference fringes. The absolute wavenumber scale of the resulting infrared absorption spectra was calibrated against the accurate CO_2_ line positions reported by Horneman.^43^ The precision of the observed line positions is estimated to be better than 0.002 cm^−1^.

## 2 Rovibrational Spectral Analysis

**Figure 1:**
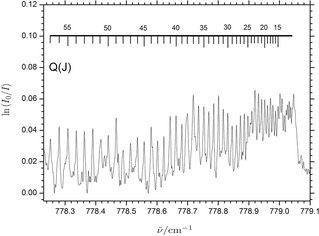
Portion of the infrared absorbance spectrum of the homodimer (HCN)_2_ showing the *ν* ${}_{6}^{1}$ band in the spectral region of the central Q‐branch. The numbered lines above the trace indicate the assigned *J*‐values for the individual rovibrational transitions (see the electronic supplemental material).

The collected infrared average absorbance spectrum is dominated by the strong degenerate bending fundamental of the HCN monomer in the entire range from 525 cm^−1^ to 835 cm^−1^. The R‐branch of this band therefore gives rise to a series of strong rovibrational lines with a spacing around 2.9 cm^−1^ in the spectral window above the HCN monomer band origin of 711.9 cm^−1^, where both the acceptor bending transition $\nu_{7}^{1}$ and the donor bending transition $\nu_{6}^{1}$ of (HCN)_2_ are expected. The collected absorbance spectrum does not show any sign of the slightly perturbed $\nu_{7}^{1}$ transition of (HCN)_2_ due to the very saturated HCN monomer absorptions in the vicinity of the band origin. However, a distinct Q‐branch structure is clearly observed around 779.05 cm^−1^ in the gap between two strong HCN monomer lines. The Q‐branch degrades towards lower energies indicating a negative value of Δ*B*=(*B*′−*B′′*) and is accompanied by weaker R‐ and P‐branches. An extensive series of more than 35 lines with a spacing around 0.10 cm^−1^ in the range between 781.4 cm^−1^ and 785.4 cm^−1^ belonging to the R‐branch is readily observable, whereas the corresponding P‐branch is severely overlapped by a second weaker Q‐branch at 777.2 cm^−1^ (not shown), which we tentatively assign to a hot band transition originating from the populated $\nu_{9}^{1}$ level. The observed rovibrational structure thus has the typical appearance of a perpendicular type band of a Σ–Π transition for a linear polyatomic molecule. Figure 1 shows the observed spectrum in the narrow region of this Q‐branch, blue‐shifted by 67.15 cm^−1^ relative to the HCN monomer fundamental, which is consequently assigned to the significantly perturbed $\nu_{6}^{1}$ transition.

The observed rotational structure of the band was analyzed employing a standard semi‐rigid polyatomic linear molecule Hamiltonian as implemented in the PGOPHER software package developed by Western^44^. This semi‐rigid linear molecule Hamiltonian is based on the rovibrational energy expressions for the vibrational ground state *E*′′ and the vibrational excited state *E*′ given in eqs. (1) and (2) including the rotational constants *B*′′ and *B*′, the quartic centrifugal distortion constants *D′′_J_* and *D′_J_* together with the *l*‐type doubling constant *q* for the degenerate level associated with the $\nu_{6}^{1}$ transition.

(1) $$E{}^{''}=B{}^{''}J\left(J+1\right)-{D{}^{''}}_{J}J^{2}{\left(J+1\right)}^{2}$$

(2) $$E{}^{'}=B{}^{'}J\left(J+1\right)-{D{}^{'}}_{J}J^{2}{\left(J+1\right)}^{2}\pm \frac{1}{2}qJ\left(J+1\right)$$

The P‐ and R‐branch transitions occur to the lower components of the doublets with an effective rotational constant (*B*′−$\frac{1}{2}$ *q*) according to the general symmetry selection rule, whereas the Q‐branch transitions occur to the upper components of the doublets with an effective rotational constant (*B*′+$\frac{1}{2}$ *q*). Separate rovibrational analyses of the Q‐branch and the P‐, R‐branch system are therefore required for the determination of *B*′ and *q*. The values of the ground‐state constants *B*” and *D”_J_* were constrained to the values reported by Larsen *et al*.^24^, based on the most comprehensive rovibrational analysis available in the literature for (HCN)_2_.

Several *J*‐assignments of the Q‐branch transitions were tested both with and without the incorporation of the *l*‐type doubling parameter. The specific assignment of the Q‐branch transitions shown in Figure 1 resulted in a significantly smaller fitting residual relative to other proposed assignments. The correct *J*‐assignments of the R‐branch transitions were rather straightforward as the unresolved beginning of the strong Q‐branch clearly indicates the origin of the band (Figure 1). A total number of 100 observed P‐ (34≤*J*′′≤45), Q‐ (13≤*J*′′≤63) and R‐transitions (20≤*J*′′≤63) were subsequently fitted simultaneously employing the Hamiltonian including the *l*‐type doubling constant and the resulting spectroscopic constants are given in Table 1. The obtained fitting residual of 0.00154 cm^−1^ is more than three times smaller than the spectral resolution as expected^10^ and the observed and calculated rovibrational transition energies are provided in the electronic supporting information.

**Table 1:** The spectroscopic constants (cm^−1^) and resulting fitting parameters obtained from the rovibrational analysis of the observed donor bending fundamental band *ν* ${}_{6}^{1}$ of (HCN)_2_.^[a]^

*ν*_0_	779.05182(50)
*B′*	0.05803055(58)
*D* ${}_{J}^{{}^{'}}$	8.151(14)⋅10^−8^
*q*	1.424(50)⋅10^−5^
*N* ^[b]^	100
*σ* ^[c]^	0.00154

^[a]^ The ground‐state constants were constrained to *B*”=0.0582570 cm^−1^ and *D”_J_*=7.745 ⋅ 10^−8^ cm^−1^.^23^ ^[b]^ Number of observations. ^[c]^ Residual of fit.

## 3 A Semi‐Experimental Vibrational Zero‐Point Energy and Dissociation Energy D_0_

The present observed *ν* ${}_{6}^{1}$ fundamental band origin provides the final significant contribution to the change of *intra*‐molecular vibrational zero‐point energy upon HCN dimerization denoted ΔZPE_intra_ as the only non‐observed *intra*‐molecular vibrational fundamental transition missing is associated with the acceptor bending mode $\nu_{7}^{1}$ , which according to quantum chemical predictions is just slightly perturbed relative to the isolated HCN monomer bending band origin.^33^, ^38^ In the discussion of how to make a reliable estimate of the total change of vibrational zero‐point energy upon dimerization ΔZPE_total_=ΔZPE_intra_+ΔZPE_inter_, where the term ΔZPE_inter_ denotes the contribution from the class of large‐amplitude *inter*‐molecular vibrational modes, we will first address the usual vibrational term values *G*(*v*) given by the second order perturbation theory expression below. For simplificity, we consider the standard vibrational term expression formulated for an asymmetric top molecule although some of these terms will be identical for a polyatomic linear molecular system as (HCN)_2_:

(3) $$G\left(v\right)=\sum_{r}\;\omega_{r}\left(v_{r}+\frac{1}{2}\right)+\sum_{r>s}\;x_{rs}\left(v_{r}+\frac{1}{2}\right)\left(v_{s}+\frac{1}{2}\right)$$

including the *r*‐th harmonic vibrational energy *ω_r_*, the vibrational anharmonicity constants *x_rs_* and the vibrational quantum number *v_r_* for the normal coordinate *r*. The “true” vibrational zero‐point energy *G*(0) for the molecule is then given by the following expression:

(4) $${\mathrm{ZPE}}_{{}^{''}true^{''}}=G\left(0\right)=\frac{1}{2}\sum_{r}\;\omega_{r}+\frac{1}{4}\sum_{r>s}\;x_{rs}=\frac{1}{2}\sum_{r}\;\omega_{r}+\frac{1}{4}\sum_{r}\;x_{rr}+\frac{1}{4}\sum_{r>s}\;x_{rs}$$

The complete set of anharmonicity constants including the diagonal terms *x_rr_* and cross‐coupling terms *x_rs_* is, however, rarely known for most molecular systems and in particular not for transient species as (HCN)_2_. We can therefore compare the expression for the “true” vibrational zero‐point energy given above with approximate expressions based solely on the sets of either harmonic vibrational energies *ω_r_* or anharmonic vibrational energies *ν_r_*, which are often more accessible. The simplest approximate expression for the vibrational zero‐point energy is found when considering solely theoretical harmonic vibrational fundamental energies:

(5) $${\mathrm{ZPE}}_{harm}^{teo}=\frac{1}{2}\sum_{r}\;\omega_{r}$$

This simple harmonic sum based on the harmonic vibrational energies shown in Table 2 clearly overestimates the “true” vibrational zero‐point energy as the difference ${\mathrm{ZPE}}_{true}-\mathrm{ZPE}$ ${}_{harm}^{teo}$ is easily seen to be $\frac{1}{4}\sum_{r}\;x_{rr}$ +$\frac{1}{4}\sum_{r>s}\;x_{rs}$ , which in general has a negative value. Alternatively, an approximate expression for the vibrational zero‐point energy based on observed (anharmonic) fundamental vibrational band origins can be considered whenever a complete set of fundamental band origins has been explored experimentally. The anharmonic vibrational energy *ν_r_* for the fundamental transition associated with the normal coordinate *r* is given by:

(6) $$\nu_{r}=G\left(v_{r}=1\right)-G\left(v_{r}=0\right)=\omega_{r}+2x_{rr}+\frac{1}{2}\sum_{r\neq s}\;x_{rs}$$

**Table 2:** The observed (anharmonic) and harmonic CCSD(T)‐F12b/aug‐cc‐pVQZ vibrational fundamental energies (cm^−1^) for the linear (HCN)_2_ homodimer classified according to the irreducible representations of the point group *C*_*∞v*_ with corresponding vibrational normal mode descriptions.

	*ν_obs_*	$\omega_{\mathrm{CCSD}(T)-F12b}$ ^[f]^	Symmetry Species	Mode Description
*ν*_1_	3308.3175(5)^[a]^	3435.0	Σ_*g*_	Acceptor CH Stretch
*ν*_2_	3241.5588(30)^[a]^	3356.5	Σ_*g*_	Donor CH Stretch
*ν*_3_	2104.6(3)^[b]^	2142.1	Σ_*g*_	Acceptor CN Stretch
*ν*_4_	2094.7(3)^[b]^	2120.8	Σ_*g*_	Donor CN Stretch
*ν*_5_	101^[c]^	118.8	Σ_*g*_	N··· HC Stretch
$\nu_{6}^{1}$	779.05182(50)^[d]^	814.2	Π	Donor HCN Bend
$\nu_{7}^{1}$	*723*(2)^[e]^	739.8	Π	Acceptor HCN Bend
$\nu_{8}^{1}$	119.11526(60)^[f]^	137.2	Π	Donor Libration
$\nu_{9}^{1}$	40.7518711(67)^[g]^	48.0	Π	Acceptor Libration

^[a]^ Jucks *et al*.^18^ ^[b]^ Maroncelli *et al*.^24^, ^25^ ^[c]^ Georgiou *et al*.^13^ ^[d]^ Present work. ^[e]^ Anharmonic prediction by Hoobler *et al*.^37^ (see text). ^[f]^ Mihrin *et al*.^32^ ^[g]^ Grushow *et al*.^26^

The approximate expression for the vibrational zero‐point energy based solely on experimental (anharmonic) fundamental band origins will therefore be given by:

(7) $${\mathrm{ZPE}}_{anh}^{obs}=\frac{1}{2}\sum_{r}\;\nu_{r}=\frac{1}{2}\sum_{r}\;\omega_{r}+\sum_{r}\;x_{rr}+\frac{1}{2}\sum_{r>s}\;x_{rs}$$

This anharmonic sum based on the observed vibrational band origins shown in Table 2 on the other hand underestimates the “true” vibrational zero‐point energy as the difference ZPE_*true*_−ZPE${}_{anh}^{obs}$ is calculated to be −$\frac{3}{4}\sum_{r}\;x_{rr}$ −$\frac{1}{4}\sum_{r>s}\;x_{rs}$ , which in general has a positive value. A robust but very simple solution to obtain a much more reliable approximation of the vibrational zero‐point energy has been demonstrated by Schaefer III *et al*.^45^ for several simple molecules as H_2_O and CH_4_, where the complete sets of anharmonicity constants are known experimentally. Schaefer III *et al*. showed that the average value of ZPE${}_{anh}^{obs}$ and ZPE${}_{harm}^{teo}$ comes very close to the “true” vibrational zero‐point energy ZPE_*true*_ and only slightly underestimate this value with a minor deviation of −$\frac{1}{4}\sum_{r}\;x_{rr}$ .

The present spectroscopic findings now enable the determination of the total change of vibrational zero‐point energy upon dimerization ΔZPE_total_=ΔZPE_intra_+ΔZPE_inter_. However, we first consider the simple approximation based on theoretical harmonic fundamental vibrational energies (eq. 5). The harmonic vibrational zero‐point energies of both (HCN)_2_ and HCN are combined for the set of intramolecular vibrational fundamental transitions including *ν*_1_, *ν*_2_, *ν*_3_, *ν*_4_, $\nu_{6}^{1}$ and $\nu_{7}^{1}$ (the mode numbering for (HCN)_2_ given in Table 2) remembering that the $\nu_{6}^{1}$ and $\nu_{7}^{1}$ fundamentals are both doubly degenerate:

(8) $$\Delta {\mathrm{ZPE}}_{harm,intra}^{teo}=\frac{1}{2}\sum_{r}\;\left(\omega_{r}^{dim}-\omega_{r}^{mon}\right)$$

The use of previously published harmonic CCSD(T)‐F12b/aug‐cc‐pVQZ intramolecular vibrational fundamental energies for both (HCN)_2_ and HCN^33^ then gives Δ${\mathrm{ZPE}}_{harm,intra}^{teo}$ =0.725 kJ mol^−1^. A harmonic contribution to the vibrational zero‐point energy Δ${\mathrm{ZPE}}_{harm,inter}^{teo}$ from the class of large‐amplitude vibrational fundamentals introduced by the complexation including *ν*_5_, $\nu_{8}^{1}$ and $\nu_{9}^{1}$ ($\nu_{8}^{1}$ and $\nu_{9}^{1}$ doubly degenerate) of 2.926 kJ mol^−1^ comes directly from eq. 5 and provides a total change of vibrational zero‐point energy in this simple harmonic approximation Δ${\mathrm{ZPE}}_{harm,total}^{teo}$ of 3.65 kJ mol^−1^ as listed in Table 3. This harmonic approximation for the total change of vibrational zero‐point energy upon dimerization clearly overestimates the “true” value as evidenced by Hoobler *et al*.’s non‐empirical theoretical prediction of 3.35 kJ mol^−1^ based on second order vibrational perturbation theory at the AE‐CCSD(T)/cc‐pCVQZ level of theory.^38^

**Table 3:** The total change of vibrational zero‐point energy (kJ mol^−1^) based on theoretical (harmonic) vibrational fundamental energies ΔZPE${}_{harm}^{teo}$ (upper limit) and observed or theoretical anharmonic fundamental energies ΔZPE_*anh*_ (lower limit). The results are compared to the recent non‐empirical benchmark values of *D_e_* and *D*_0_ by Hoobler *et al*.^38^

	*D_e_*	ΔZPE${}_{harm}^{teo}$	ΔZPE${}_{anh}$	ΔZPE_*best*_	*D*_0_
Present work (semi‐exp)	19.83^[a]^	3.65^[b]^	3.05^[c]^	3.35^[d]^	16.48
Hoobler *et al*. (teo)	19.83^[a]^		3.03^[e]^	3.35^[f]^	16.48

^[a]^ AE‐CCSDT(Q)/CBS value including relativistic and diagonal Born‐Oppenheimer terms by Hoobler *et al*.^37^ ^[b]^ Based on harmonic predictions at the CCSD(T)‐F12b/aug‐cc‐pVQZ level of theory by Mihrin *et al*.^32^ ^[c]^ Based on observed (anharmonic) fundamental band origins (see text). ^[d]^ The average value of ΔZPE${}_{harm}^{teo}$ and ΔZPE${}_{anh}^{obs}$ (see text). ^[e]^ Based on theoretical anharmonic fundamental band origins. ^[f]^ Second order vibrational perturbation theory (VPT2) at the AE‐CCSD(T)/cc‐pCVQZ level of theory.

In our similar approach to obtain the anharmonic change of vibrational zero‐point energy Δ${\mathrm{ZPE}}_{anh,total}^{obs}$ based on the almost complete set of observed fundamental band origins (eq. 7), we first discuss shortly the expected minor contribution from the intramolecular acceptor bending fundamental $\nu_{7}^{1}$ , which has not been observed in the gas‐phase. The present sensitive long‐path FTIR synchrotron spectroscopy approach does not reveal any signs of this transition owing to severe spectral overlaps with monomeric HCN absorption in the vicinity of the degenerate bending fundamental band origin of 711.90 cm^−1^ for HCN. This suggests that the $\nu_{7}^{1}$ fundamental transition indeed is only very slightly blue‐shifted relative to the monomer as predicted by both harmonic (dimerization shift of 12 cm^−1^)^33^ and anharmonic (dimerization shift of 11 cm^−1^)^38^ vibrational force field calculations. In general, Hoobler *et al*.’s recent anharmonic predictions reproduce the observed intramolecular complexation shifts rather well with the largest error of 9.15 cm^−1^ for the donor bending fundamental $\nu_{6}^{1}$ reported in the present investigation (predicted blue‐shift of 58 cm^−1^ versus observed blue‐shift of 67.15 cm^−1^). Assuming a similar maximum relative error for the predicted dimerization shift for the intramolecular acceptor bending $\nu_{7}^{1}$ fundamental, we can safely estimate a dimerization blue‐shift of 11(2) cm^−1^ and thereby a band origin of 723(2) cm^−1^ as given in Table 2.

The approximate anharmonic change of vibrational zero‐point energy based on the set of observed fundamental transition energies according to eq. 7 then gives Δ${\mathrm{ZPE}}_{anh,intra}^{obs}$ =0.535 kJ mol^−1^ and Δ${\mathrm{ZPE}}_{anh,inter}^{obs}$ =2.515 kJ mol^−1^ and a resulting value for Δ${\mathrm{ZPE}}_{anh,total}^{obs}$ of 3.05 kJ mol^−1^ as listed in Table 3. This approximate value is a lower limit for the “true” total change of vibrational zero‐point energy as argued for above and again indicated by the 0.3 kJ mol^−1^ higher value suggested by Hoobler *et al*.^37^ The best semi‐experimental value for the “true” change of vibrational zero‐point energy is then achieved by computing the average value of Δ${\mathrm{ZPE}}_{harm,total}^{teo}$ and Δ${\mathrm{ZPE}}_{anh,total}^{obs}$ although this average value still underestimates the “true” value slightly. It then appears that our best semi‐experimental value matches spot on Hoobler *et al*.’s non‐empirical value of 3.35 kJ mol^−1^ (Table 3).

The immediate remarkable correspondence between our semi‐experimental value and Hoobler *et al*.’s entirely theoretical value is rather surprising as the application of second order vibrational perturbation theory is known to be notoriously challenging for weakly bound molecular systems. As with any other perturbational approach, one should be careful if the perturbations, the anharmonicity contributions, are of considerable magnitude relative to the zeroth‐order harmonic vibrational energy references. The class of large‐amplitude intermolecular vibrational modes in particular may be problematic for this kind of perturbational approach, where Hoobler *et al*. reported anharmonicity contributions in the order of 12 % and 24 % for the $\nu_{8}^{1}$ and $\nu_{9}^{1}$ fundamentals, respectively. Hoobler *et al*.’s anharmonic analysis showed no Fermi resonance type terms based upon near degeneracy between fundamental transitions and vibrational hot band transitions or strong interactions observed as large cubic force constants. They tested, however, extensively their results by the removal of one or more of the large‐amplitude vibrational modes *ν*_5_, $\nu_{8}^{1}$ and $\nu_{9}^{1}$ from the anharmonic perturbational treatment. The removal of the $\nu_{8}^{1}$ fundamental alone in the anharmonic analysis seemed to produce the most significant effects in the predicted anharmonic band origins, as the $\nu_{9}^{1}$ band origin shifts from 35 cm^−1^ to 26 cm^−1^ and the $\nu_{8}^{1}$ band origin shifts from 120 cm^−1^ to 136 cm^−1^.

The complete sets of theoretical anharmonic fundamental band origins for both HCN and (HCN)_2_ reported by Hoobler *et al*. enables us to calculate the approximate (lower limit) of the total change of vibrational zero‐point energy based on eq. 7 ignoring all involved anharmonicity constants. The extracted value of 3.03 kJ mol^−1^ (Table 3) again is remarkable close to the value based on the complete set of experimental (anharmonic) fundamental band origins of 3.05 kJ mol^−1^. A closer look at Hoobler *et al*.’s reported non‐empirical anharmonic band positions, however, reveals total numeric deviations $\sum_{r}\;|$ $\nu_{r}^{VPT2}-\nu_{r}^{obs}$ | in the order of 10 cm^−1^ for the HCN monomer and 44.7 cm^−1^ for the (HCN)_2_ system. These deviations would potentially introduce an error of 0.4 kJ mol^−1^ in this approximate value for the total vibrational zero‐point energy Δ${\mathrm{ZPE}}_{anh,total}$ if the theoretical anharmonic band origins systematically were predicted at higher energies than the observed band origins. The excellent agreement between the observed and theoretical values of ${\Delta \mathrm{ZPE}}_{anh,total}$ is partly due to cancellation of errors with opposite signs, where overestimated fundamental band origins are canceled out by underestimated fundamental band positions of the same order of magnitude. Nevertheless, the present semi‐experimental approach for the determination of the total change of vibrational zero‐point energy upon (HCN)_2_ complexation combined with the AE‐CCSDT(Q)/CBS benchmark *D_e_*‐value by Hoobler *et al* then reproduces the “best” value of 16.48 kJ mol^−1^ for the dissociation energy *D*_0_ (Table 3). Based on our estimated lower (anharmonic) and higher (harmonic) limits for the “true” value of ΔZPE_*total*_, we bracket this final semi‐empirical dissociation energy with an error of ±0.30 kJ mol^−1^, which should even include minor remaining errors of the benchmark electronic energies (±0.05 kJ mol^−1^) as suggested by Hoobler *et al*. The present “best” semi‐experimental estimate of *D*_0_ is slightly lower than the value of 17.2 kJ mol^−1^ estimated previously by the same authors based on the observed change of vibrational zero‐point energy from the class of large‐amplitude vibrational modes alone.^33^ The previous semi‐experimental estimate of *D*_0_ employed the harmonic prediction of the intramolecular donor bending $\nu_{6}^{1}$ fundamental band origin now available by experiment. However, more importantly the present work now employs Schaefer *et* *al*.’s accurate approach to extract the “true” intermolecular vibrational zero‐point energy contribution by considering an average of the theoretical harmonic and experimental (anharmonic) vibrational fundamental energies.^45^

## 4 Conclusions

In summary, the generated high‐resolution synchrotron infrared absorption spectrum of (HCN)_2_ has enabled a detailed rovibrational analysis of the missing $\nu_{6}^{1}$ band associated with the doubly degenerate donor bending mode. The observed rovibrational structure has the characteristics of a perpendicular type band of a Σ−Π transition for a linear polyatomic molecule and in total 100 spectral lines belonging to the P‐, Q‐, and R‐branches have been assigned and fitted simultaneously to a standard semi‐rigid linear molecule Hamiltonian. The resulting fit provides an accurate value for the missing band origin ν_0_ of 779.05182(50) cm^−1^ together with the spectroscopic parameters *B*’, *D*’_*J*_ and *q* for the doubly degenerate excited state $\nu_{6}^{1}$ . This accurate $\nu_{6}^{1}$ fundamental band origin, blue‐shifted by 67.15 cm^−1^ relative to the degenerate HCN monomer bending band origin, provides the final significant contribution of 0.8 kJ mol^−1^ to the change of *intra*‐molecular vibrational zero‐point energy upon complexation and the best semi‐experimental estimate of 3.35±0.30 kJ mol^−1^ for the total change of vibrational zero‐point energy. The combination with Hoobler *et* *al*.’s^37^ AE‐CCSDT(Q)/CBS benchmark value for the interaction energy *D*_e_ of 19.83 kJ mol^−1^ including relativistic and diagonal Born‐Oppenheimer corrections then enables a reliable semi‐experimental value of 16.48±0.30 kJ mol^−1^ for the intermolecular hydrogen bond energy *D*_0_.

## Supporting Information

The observed and calculated line positions from the rovibrational analysis of the $\nu_{6}^{1}$ band are given as electronic supporting information.

## Conflict of interest

The authors declare no conflict of interest.

## Supporting information

As a service to our authors and readers, this journal provides supporting information supplied by the authors. Such materials are peer reviewed and may be re‐organized for online delivery, but are not copy‐edited or typeset. Technical support issues arising from supporting information (other than missing files) should be addressed to the authors.

Supplementary
