# Supplementary material for: High‐Resolution Infrared Synchrotron Investigation of (HCN)2 and a Semi‐Experimental Determination of the Dissociation Energy D 0
Source: Chemphyschem. 2019 Nov 8;20(23):3238–44. doi: 10.1002/cphc.201900811 (PMC6916300; doi:10.1002/cphc.201900811)
Supplement: Supplementary file 1 — Supplementary [file CPHC-20-3238-s001.pdf]

CHEMPHYSCHEM

Supporting Information

© Copyright Wiley-VCH Verlag GmbH & Co. KGaA, 69451 Weinheim, 2019

High-Resolution Infrared Synchrotron Investigation of $(HCN)_2$ and a Semi-Experimental Determination of the Dissociation Energy D_0

D. Mihrin, P. W. Jakobsen, A. Voute, L. Manceron, and R. Wugt Larsen*

Table S1: The Observed and Calculated Rovibrational Line Positions with Deviations and the Corresponding Assignments for the Observed Donor Bending Band ν_6^1 of $(HCN)_2$.

Observed (cm^{-1})	Calculated (cm^{-1})	Obs-Calc (cm^{-1})	Assignment
773.378	773.3808	-0.0029	P(45)
773.5161	773.5167	-0.0006	P(44)
774.0551	774.0554	-0.0003	P(40)
774.1885	774.1889	-0.0003	P(39)
774.3221	774.3219	0.0002	P(38)
774.4552	774.4545	0.0007	P(37)
774.587	774.5866	0.0004	P(36)
774.7188	774.7182	0.0006	P(35)
774.8507	774.8494	0.0013	P(34)
778.066	778.0687	-0.0026	Q(63)
778.0988	778.1006	-0.0018	Q(62)
778.1313	778.132	-0.0007	Q(61)
778.1615	778.1628	-0.0014	Q(60)
778.1923	778.1931	-0.0008	Q(59)
778.2231	778.2227	0.0004	Q(58)
778.2524	778.2518	0.0006	Q(57)
778.2806	778.2803	0.0003	Q(56)
778.3094	778.3083	0.0011	Q(55)
778.3372	778.3357	0.0016	Q(54)
778.3636	778.3625	0.0011	Q(53)
778.39	778.3888	0.0012	Q(52)
778.4157	778.4145	0.0012	Q(51)
778.4407	778.4397	0.0010	Q(50)
778.4667	778.4643	0.0024	Q(49)
778.4891	778.4884	0.0008	Q(48)
778.5138	778.5119	0.0019	Q(47)
778.5364	778.5349	0.0014	Q(46)
778.5584	778.5574	0.0010	Q(45)
778.5812	778.5794	0.0019	Q(44)
778.6019	778.6008	0.0012	Q(43)
778.6222	778.6217	0.0006	Q(42)
778.6429	778.6421	0.0008	Q(41)
778.662	778.6619	0.0001	Q(40)
778.6812	778.6813	0.0000	Q(39)
778.6988	778.7001	-0.0013	Q(38)
778.7186	778.7184	0.0002	Q(37)
778.7358	778.7362	-0.0004	Q(36)
778.7528	778.7535	-0.0007	Q(35)
778.7691	778.7703	-0.0011	Q(34)
778.7854	778.7865	-0.0011	Q(33)
778.8012	778.8023	-0.0012	Q(32)

778.8164	778.8176	-0.0012	Q(31)
778.8306	778.8324	-0.0017	Q(30)
778.8451	778.8467	-0.0016	Q(29)
778.8593	778.8605	-0.0012	Q(28)
778.8721	778.8738	-0.0017	Q(27)
778.8846	778.8866	-0.0020	Q(26)
778.8966	778.8989	-0.0023	Q(25)
778.9098	778.9108	-0.0010	Q(24)
778.9198	778.9221	-0.0023	Q(23)
778.9311	778.933	-0.0019	Q(22)
778.9411	778.9434	-0.0022	Q(21)
778.9517	778.9533	-0.0015	Q(20)
778.9612	778.9627	-0.0015	Q(19)
778.9707	778.9716	-0.0009	Q(18)
778.9788	778.9801	-0.0013	Q(17)
778.9876	778.9881	-0.0005	Q(16)
778.9956	778.9956	0.0000	Q(15)
779.0021	779.0027	-0.0006	Q(14)
779.0088	779.0092	-0.0004	Q(13)
781.3969	781.3932	0.0037	R(20)
781.5032	781.4994	0.0037	R(21)
781.6086	781.6051	0.0035	R(22)
781.7139	781.7103	0.0037	R(23)
781.8175	781.8149	0.0026	R(24)
781.9221	781.919	0.0031	R(25)
782.0257	782.0226	0.0031	R(26)
782.1268	782.1256	0.0012	R(27)
782.2302	782.2281	0.0020	R(28)
782.3317	782.3301	0.0016	R(29)
782.4333	782.4315	0.0019	R(30)
782.7321	782.7322	-0.0001	R(33)
782.8322	782.8313	0.0009	R(34)
782.9295	782.9298	-0.0003	R(35)
783.0274	783.0278	-0.0004	R(36)
783.1252	783.1251	0.0001	R(37)
783.2209	783.2218	-0.0008	R(38)
783.3175	783.3179	-0.0004	R(39)
783.412	783.4133	-0.0013	R(40)
783.5074	783.5082	-0.0007	R(41)
783.6011	783.6024	-0.0012	R(42)
783.6943	783.6959	-0.0016	R(43)
783.7868	783.7888	-0.0020	R(44)
783.9719	783.9726	-0.0007	R(46)
784.0615	784.0635	-0.0020	R(47)
784.1525	784.1538	-0.0013	R(48)

784.2427	784.2433	-0.0006	R(49)
784.4194	784.4202	-0.0008	R(51)
784.5061	784.5076	-0.0015	R(52)
784.5938	784.5942	-0.0004	R(53)
784.6797	784.6801	-0.0004	R(54)
784.764	784.7653	-0.0014	R(55)
784.8499	784.8497	0.0002	R(56)
784.9329	784.9334	-0.0005	R(57)
785.0169	785.0163	0.0007	R(58)
785.0983	785.0983	0.0000	R(59)
785.1814	785.1796	0.0018	R(60)
785.2616	785.2601	0.0015	R(61)
785.3418	785.3398	0.0020	R(62)
785.419	785.4186	0.0003	R(63)
